# Supplementary material for: Assessment of Uptake, Accumulation and Degradation of Paracetamol in Spinach (Spinacia oleracea L.) under Controlled Laboratory Conditions
Source: Plants (Basel). 2022 Jun 21;11(13):1626. doi: 10.3390/plants11131626 (PMC9269112; doi:10.3390/plants11131626)
Supplement: Supplementary file 1 [file plants-11-01626-s001.zip › Supplementary file- Revised V2.pdf]

plants

Assessment of Uptake, Accumulation and Degradation of Paraceta-mol in Spinach (*Spinacia oleracea* L.) under Controlled Laboratory Conditions

Supplementary Table S1. Results of one-way ANOVAs showing the effect of different paracetamol treatments on the morphological attributes of spinach after 8 days.

Morphological attributes	df	F	P
Shoot length	3	45.33	<0.001
Root length	3	10.90	<0.01
Leaf number	3	4.85	<0.05
Growth tolerance index of shoots (GTIS)	2	74.87	<0.001
Growth tolerance index of roots (GTIR)	2	1.20	ns

ns: non-significant at $p \leq 0.05$.

Supplementary Table S2. Results of one-way ANOVAs showing the effect of different paracetamol treatments on the concentration (mg/kg DW) of several macronutrients, micronutrients and sodium in spinach shoots and roots after 8 days.

Elements in spinach shoots (mg/kg DW)	df	F-Ratio	p-Value
Ca	3	72.44	<0.001
K	3	21.69	<0.001
Mg	3	276.93	<0.001
Fe	3	186.86	<0.001
Mn	3	4.45	<0.05
Na	3	5.65	<0.001
**Elements in spinach roots (mg/kg DW)**	**df**	**F-Ratio**	**p-Value**
Ca	3	6.43	<0.05
K	3	26.32	<0.001
Mg	3	7.70	<0.01
Fe	3	13.08	<0.05
Mn	3	5.61	<0.05
Na	3	1.52	ns

ns: non-significant at $p \leq 0.05$.

Supplementary Table S3. Results of One-way ANOVAs showing the effect of different paracetamol treatments on C, H, N, and S percentage in spinach.

Elements	df	F	P
C	3	1.38	ns
H	3	0.68	ns
N	3	22.91	<0.001
S	3	7.50	<0.05

ns: non-significant at $p \leq 0.05$

Supplementary Table S4. Gradient separation method for LC-MS analysis of paracetamol.

Time(min)	Flow rate ml/min	A%	B%
Initial	0.3	95	5
0.5	0.3	95	5
3.5	0.3	50	50
5.5	0.3	30	70
6	0.3	95	5

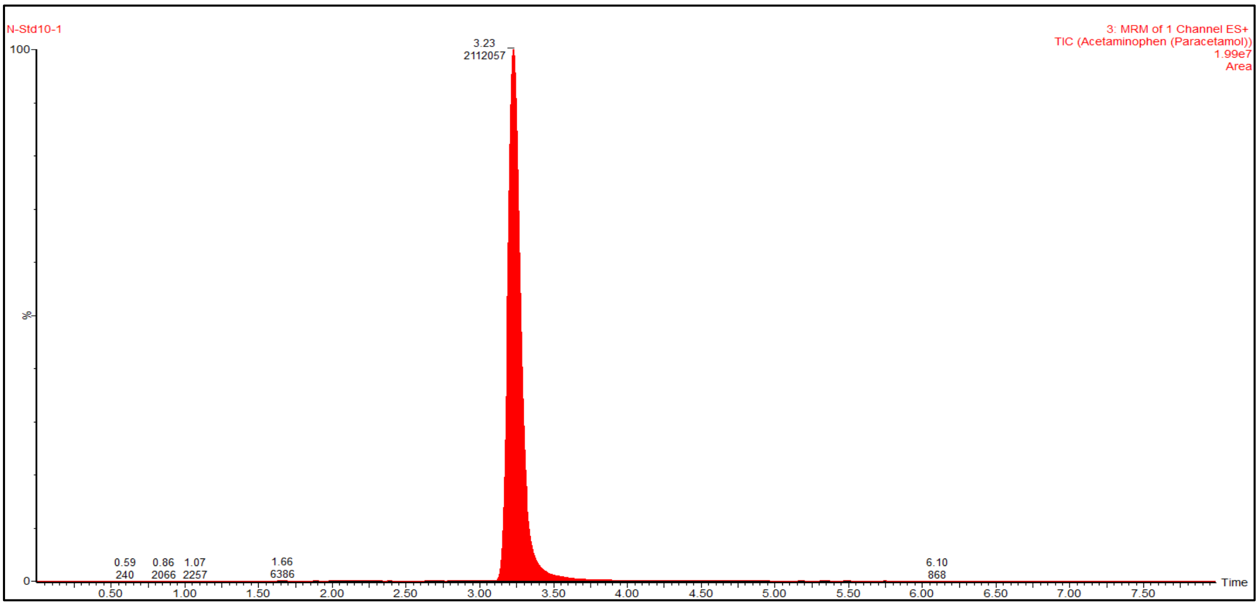

Supplementary Figure S1. LC/MS chromatogram of Paracetamol (Acetaminophen) standard.

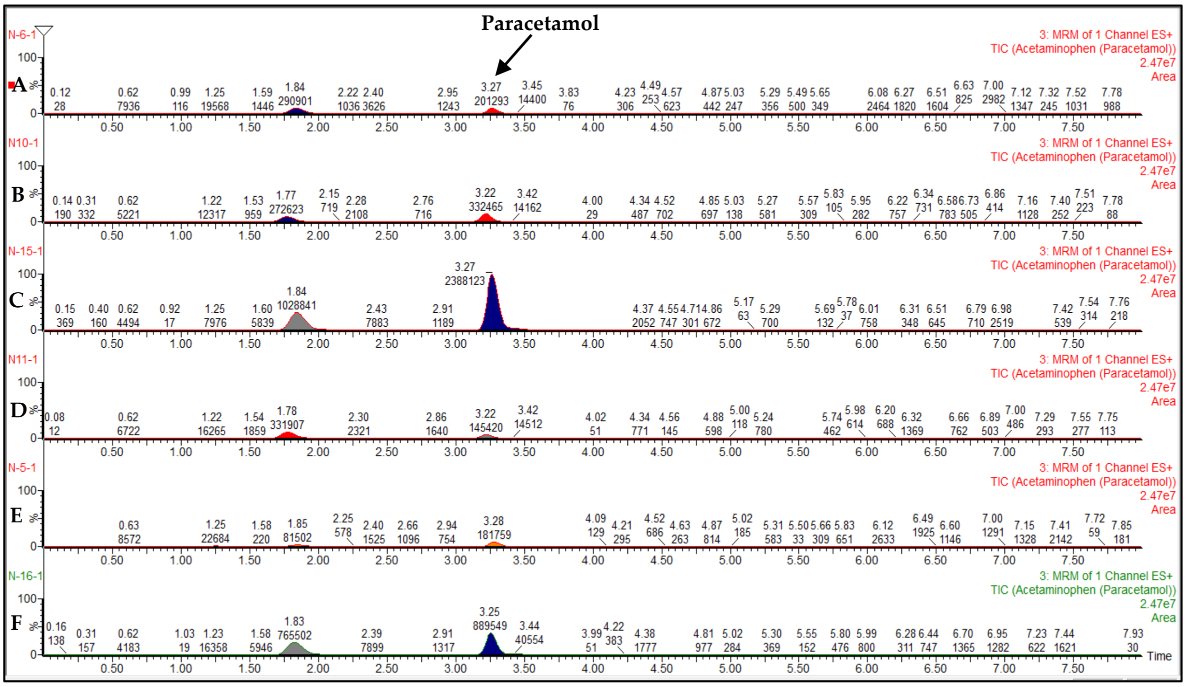

Supplementary Figure S2. LC/MS chromatograms showing the concentration of paracetamol (Retention time: 3.2 min) in spinach shoots for (A) 50 mg/L, (B) 100 mg/L and (C) 200 mg/L paracetamol treatments after 4 days, and for (D) 50 mg/L, (E) 100 mg/L and (F) 200 mg/L paracetamol treatments after 8 days.

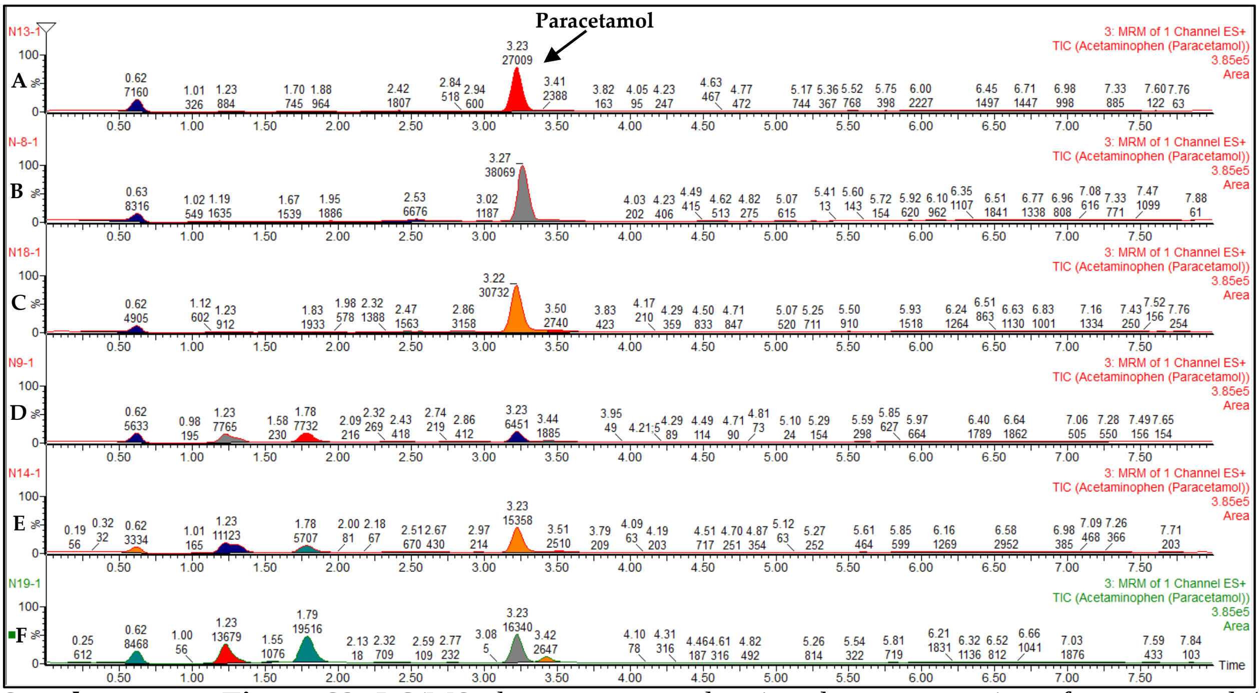

Supplementary Figure S3. LC/MS chromatograms showing the concentration of paracetamol (Retention time: 3.2 min) in spinach roots for (A) 50 mg/L, (C) 100 mg/L and (B) 200 mg/L paracetamol treatments after 4 days, and for (D) 50 mg/L, (E) 100 mg/L and (F) 200 mg/L paracetamol treatments after 8 days.

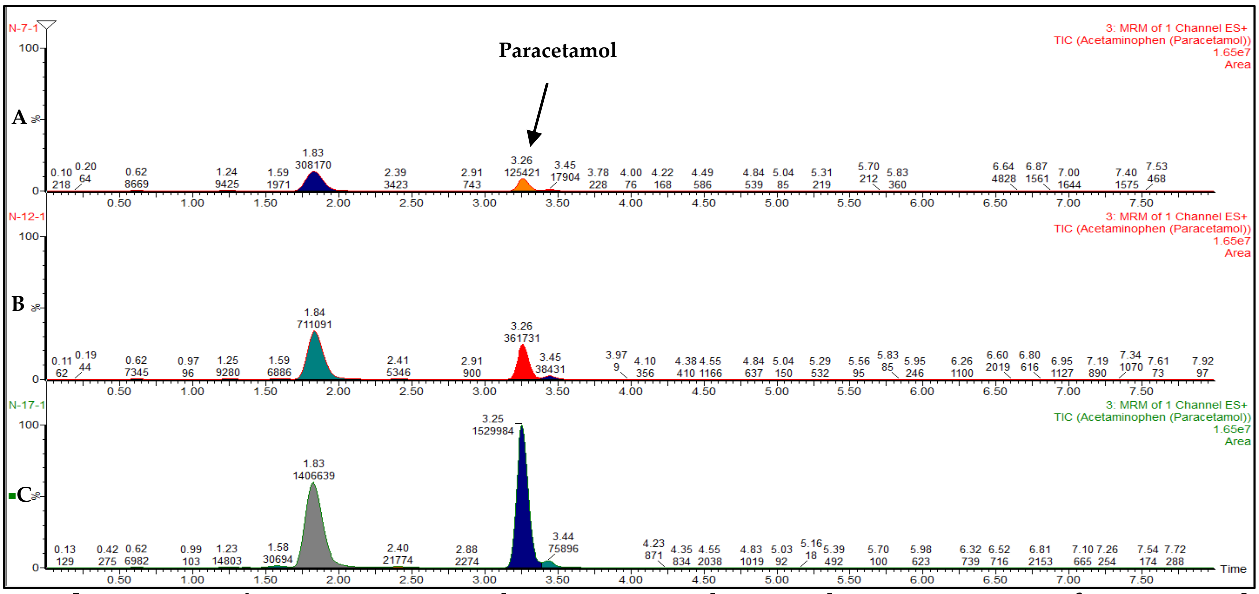

Supplementary Figure S4. LC/MS chromatograms showing the concentration of paracetamol (Retention time: 3.2 min) in spinach dry leaves for (A) 50 mg/L, (B) 100 mg/L, and (C) 200 mg/L paracetamol treatments after 8 days.

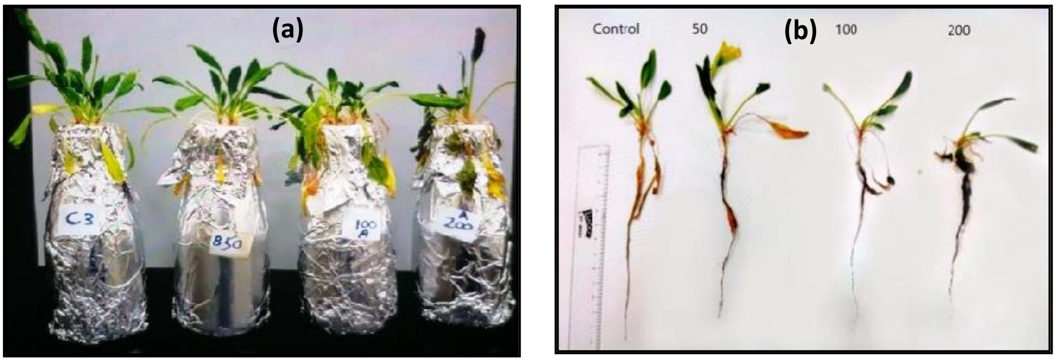

Supplementary Figure S5. Images of untreated (control) and treated spinach plants with 50 mg/L, 100 mg/L, and 200 mg/L paracetamol (a,b).
